# Dogs and cats with acute-on-chronic kidney disease have worse outcomes than those with acute kidney injury following renal replacement therapy

**DOI:** 10.3389/fvets.2026.1857111

**Published:** 2026-07-30

**Authors:** Woonchan Ahn, Keon Kim, Jinhae Kim, Yujin Lee, Taeho Lee, Jiwoong Her

**Affiliations:** 1 Signature Animal Medical Center, Seoul, Republic of Korea; 2 Department of Veterinary Internal Medicine and Institute of Veterinary Science, College of Veterinary Medicine, Kangwon National University, Chuncheon-si, Gangwon-do, Republic of Korea; 3 Department of Veterinary Clinical Sciences, College of Veterinary Medicine, North Carolina State University, Raleigh, NC, United States; 4 Laboratory of Veterinary Internal Medicine, College of Veterinary Medicine and Veterinary Medical Research Institute, Jeju National University, Jeju, Republic of Korea; 5 Veterinary Emergency Medicine, Department of Veterinary Clinical Science, College of Veterinary Medicine, Research Institute for Veterinary Science, Seoul National University, Seoul, Republic of Korea

**Keywords:** acute kidney injury, acute-on-chronic kidney disease, cat, chronic kidney disease, dog, hemodialysis, renal replacement therapy

## Abstract

**Objective:**

To compare survival to discharge and long-term outcomes in dogs and cats with acute-on-chronic kidney disease (ACKD) compared to acute kidney injury (AKI) treated with renal replacement therapy (RRT) and to identify factors associated with outcome.

**Design:**

Retrospective observational design.

**Setting:**

Single referral hospital.

**Animals:**

136 client-owned animals (67 dogs and 69 cats) treated with RRT between 2017 and 2024.

**Measurements and main results:**

This retrospective observational cohort study reviewed medical records from a referral hospital. Animals were classified as having ACKD (*n* = 66) or AKI (*n* = 70). Short-term survival was evaluated as survival to discharge, restricted mean survival time (RMST) and logistic regression models. Long-term survival was assessed using Kaplan–Meier analysis, RMST and Cox proportional hazards models. Animals with ACKD were older than those with AKI (10.4 ± 3.9 years vs. 6.3 ± 3.3 years; *p* < 0.0001) and had higher serum creatinine concentrations at discharge (3.38 ± 1.16 mg/dL vs. 2.81 ± 2.92 mg/dL; adjusted *p* = 0.0073). Survival to discharge was 53% (35/66) for ACKD and 70% (49/70) for AKI. Short-term survival did not differ significantly between groups (log-rank *p* = 0.089). Among animals survived to discharge, RMST was shorter for ACKD. Median survival time for the ACKD group was 57 days overall and 33 days for dogs.

**Conclusion:**

Although statistically significant differences were not identified, dogs and cats with ACKD had lower survival to discharge and numerically shorter long-term survival than animals with AKI. Nevertheless, 53% of RRT-treated animals with ACKD survived to discharge, demonstrating that short-term survival is achievable in selected ACKD patients.

## Introduction

Acute kidney injury (AKI) and chronic kidney disease (CKD) are major causes of morbidity and mortality in dogs and cats (1, 2). Over the past few decades, advances in veterinary medicine have substantially improved the management of patients with severe kidney disease (3). In particular, the introduction and wider availability of renal replacement therapy (RRT), has expanded the window of treatment as a life-saving option for dogs and cats with severe uremia that do not respond to conventional medical treatment (3, 4).

In clinical practice, veterinarians frequently encounter not only “pure” AKI in previously healthy animals, but also acute-on-chronic kidney disease (ACKD), in which an acute insult is superimposed on pre-existing CKD. Acute kidney injury is potentially reversible, as renal function may recover through regeneration once the underlying cause is treated and appropriate management is instituted on time, whereas CKD is characterized by irreversible structural damage and loss of functional reserve. Consequently, dogs and cats with ACKD, in whom these two pathophysiologic processes coexist, may follow a different clinical course from those with *de novo* AKI (2, 4).

Two recent veterinary studies on ACKD have reported comparable short-term outcome to AKI patients (5, 6). However, most dogs and cats in those studies recovered with medical management without hemodialysis, highlighting the need for data on patients that require hemodialysis. In cases severe enough to necessitate RRT, there is a concern that the exhausted renal reserve in CKD patients may reduce their resilience, together with complications associated with severe illness, may lead to worse prognosis than in patients with *de novo* AKI.

Therefore, the aim of this study was to compare short-term (survival to discharge) and long-term survival (post-survival discharge) between dogs and cats with AKI and those with ACKD treated with RRT. We hypothesized that, among severely azotemic patients requiring RRT, animals with ACKD would have similar survival to discharge compared with those with AKI, but poorer long-term outcomes after discharge. In addition, this study sought to identify risk factors associated with survival, in order to support clinical decision-making and prognostication for patients with ACKD requiring RRT.

## Materials and methods

This is a retrospective observational cohort study. Medical records from a single referral veterinary hospital were reviewed for dogs and cats diagnosed with AKI or ACKD 2017 and 2024. From these cases, only patients that received hemodialysis were included. Extracted data comprised signalment (age, sex, body weight, and species), serum creatinine, blood urea nitrogen (BUN) and phosphate concentration at presentation, serum creatinine concentration at discharge, Urine output at presentation, length of hospitalization, total number of dialysis sessions, etiology of AKI status, in-hospital mortality, post-discharge follow-up and survival status. Urine output was categorized as follows: anuria (<0.1 mL/kg/h), oliguria (0.1–1 mL/kg/h), normal urine output (1–2 mL/kg/h), and polyuria (>2 mL/kg/h) (7). Segev model C score (8) was calculated based on retrospective review of the medical records. Details pertaining to the duration of RRT and the urea reduction ratio for each session, up to the fifth session, were reviewed.

All dogs and cats were eligible for inclusion if they were admitted to the veterinary hospital with an acute onset of clinical signs consistent with AKI, in accordance with the diagnostic criteria of the IRIS AKI guidelines (3). The ACKD group was defined using criteria adapted from two previous studies on ACKD in dogs and cats (5, 6). For dogs, patients were assigned to the ACKD group if they fulfilled at least one of the following indicators of underlying CKD: (a) a documented prior diagnosis of CKD based on persistently increased serum creatinine, with a concurrent rise in serum creatinine of >25% above the previously recorded baseline; (b) historical laboratory evidence of chronic renal parenchymal injury obtained prior to the acute episode (when available), such as persistent renal proteinuria or clinically significant cylindruria, without concurrent baseline azotemia; or (c) abdominal ultrasonographic changes compatible with CKD, defined by the presence of ≥2 of the following: increased renal echogenicity, markedly reduced corticomedullary distinction, decreased or asymmetric kidney size, or renal cysts/irregular renal contour. For cats, patients were classified as ACKD if at least one of the following criteria was met: (1) a previous diagnosis of CKD with a concurrent increase in serum creatinine >20% above the most recent measurement; (2) a previous diagnosis of CKD together with urinary findings indicative of acute tubular injury (e.g., glucosuria with normoglycemia, cylindruria); or (3) unequivocal ultrasonographic evidence of CKD, defined by ≥2 of the following: increased cortical echogenicity, markedly diminished corticomedullary differentiation, reduced kidney size (<2.8 cm), renal cysts, or irregular renal contour. In both species, animals that did not fulfill these CKD criteria were categorized as belonging to the AKI group. Among animals classified as AKI or ACKD, RRT was performed when extracorporeal support was considered clinically indicated based on the overall clinical assessment. Indications included severe azotemia or uremic signs, fluid overload or oliguria/anuria, acid–base abnormalities, and severe electrolyte derangements when present. Uncontrolled electrolyte imbalance was not the primary indication for RRT in all cases. Dehydration and hypovolemia were assessed and optimized before RRT when clinically feasible. Many animals had already received fluid therapy before referral, whereas some were presented with fluid overload, oliguria, or anuria; therefore, the decision to initiate RRT was based on the overall assessment of volume status and clinical stability.

In general, RRT was delivered using continuous venovenous hemodiafiltration or continuous venovenous hemodialysis modes on CRRT machines (PrisMax, Baxter International Inc.; MultiFiltrate, Fresenius Medical Care) with an extracorporeal circuit incorporating an integrated hemofilter (HF20 set; FMC Paed and Midi) over 4–8 h. The ultrafiltration rate was determined based on hydration status, anticipated tolerance to fluid removal, and the resulting filtration fraction, with the goal of avoiding increases in filtration fraction above 20%. Circuits were primed primarily with 0.9% saline; however, when circuit volume exceeded 20% of the dog’s estimated blood volume, the circuit was primed with whole blood. Anticoagulation was achieved using systemic heparinization in all cases. Anticoagulation was monitored using activated partial thromboplastin time (aPTT) in earlier cases and activated clotting time (ACT) after ACT monitoring became available, with a target of approximately 1.5–2 times the baseline clotting time when feasible. The extracorporeal circuit was also visually monitored for clot formation, and patients were monitored for hemorrhage.

Following the etiologic categorization used in previous ACKD studies in dogs and cats, presumptive etiologies were classified as inflammatory, obstructive, ischemic, pyelonephritis, toxic, other renal-related, or unknown (5, 6). Inflammatory causes included pancreatitis, diagnosed based on compatible history, clinical signs, ultrasonographic findings, and increased quantitative serum canine pancreatic lipase concentration; and glomerulopathies, defined by persistent, high-grade proteinuria after exclusion of prerenal and postrenal causes. Ureteral obstruction was diagnosed when patients exhibited compatible clinical signs together with ultrasonographic evidence. Renal ischemia was considered the underlying cause in patients that had received furosemide or nonsteroidal anti-inflammatory drugs (NSAIDs), in those in which the acute episode developed after general anesthesia, in animals with clinical signs of severe dehydration, or in those with a history of traumatic injury. Similar to the previous ACKD studies, furosemide and NSAIDs associated cases were included in the ischemic category when the clinical context suggested reduced renal perfusion or hemodynamic renal injury (9–11). Pyelonephritis was suspected when a positive urine culture and pyuria on urinalysis were present, along with supportive ultrasonographic findings. “Other cause” included cardiorenal syndrome and polycystic kidney disease. A presumptive diagnosis of toxicity was made when there was a clear history of exposure to a specific toxin accompanied by compatible clinical signs, hematologic abnormalities, and ultrasonographic changes. When two or more presumptive causes were identified, the primary cause was selected for statistical analysis. Cases that did not fulfill criteria for any of the defined categories were classified as having an unknown etiology.

Long-term outcome for animals that survived to discharge was defined as the number of days from hospital discharge to death or euthanasia from any cause. Dates of death or euthanasia were obtained from medical records. Animals for which outcome data were unavailable were censored from the analysis. Therefore, survival analyses reflected all-cause mortality rather than kidney disease-specific mortality.

### Statistical analysis

All statistical analyses were performed using R software (version 4.3.2; R Foundation for Statistical Computing, Vienna, Austria). For the overall cohort, comparative analyses were conducted between the ACKD and AKI groups. Within each group, categorical variables were summarized as counts (*n*), and most continuous variables were summarized as mean (SD). As exceptions, the number of hemodialysis sessions was summarized as median (Q1, Q3), and length of hospitalization was summarized as both mean (SD) and median (Q1, Q3) (SD = standard deviation; Q1/Q3 = 25th/75th percentiles).

Differences between the ACKD and AKI groups were evaluated for age, body weight, creatinine at presentation, creatinine at discharge, blood urea nitrogen (BUN) at presentation, phosphorus at presentation, Segev model C, length of hospitalization, and number of hemodialysis during hospitalization. The normality assumption for each variable within each group was first assessed using the Shapiro–Wilk test. When the Shapiro–Wilk test yielded a *p*-value <0.05, the normality assumption was considered violated, and a nonparametric two-sample test (Wilcoxon rank-sum test) was used for between-group comparisons. Conversely, when both groups had Shapiro–Wilk test *p*-values >0.05, a two-sample *t*-test was applied. Because multiple variables were tested, *p*-values were adjusted using the Bonferroni correction to control the family-wise type I error rate. Fisher’s exact test was used to evaluate the association between etiology and disease group in the overall cohort. Heatmaps were used to summarize the number of cases in each group.

Survival to discharge was compared between the ACKD and AKI groups. Kaplan–Meier survival curves and log-rank tests were used to compare short-term outcomes during hospitalization between the ACKD and AKI groups. To examine the association between each predictor variable and survival at discharge, univariable Firth penalized logistic regression was performed, with survival coded as event = 1 in R software. Each model was specified as follows: $\text{logit}\{P(Y=1)\}=\beta_{0}+\beta_{1}X$ where Y denotes survival status and X denotes a single predictor variable entered individually. Odds ratios (ORs) were reported to quantify unadjusted associations. Natural log transformations were applied to creatinine and BUN to improve model fit. Variables with a univariable *p*-value <0.20 were selected for inclusion in the subsequent multivariable Firth penalized logistic regression model; sex was excluded because of model instability related to its multiple categorical levels.

Animals that survived to hospital discharge were included in the evaluation of post-discharge long-term survival, with the date of discharge defined as time 0. Death was coded as the event of interest, and animals that were alive at the last follow-up were treated as right-censored. Kaplan–Meier survival curves and log-rank tests were used to compare post-discharge long-term outcomes between the ACKD and AKI. Restricted mean survival time (RMST) was reported for each cohort. For RMST, Wald-type 95% confidence intervals were calculated based on the standard error of the RMST estimate. Median survival time could not be estimated for some cohorts because the survival probability did not fall below 50% in the dataset.

To investigate the unadjusted association between each candidate predictor and long-term survival, univariable Firth-penalized Cox proportional hazards models were first fitted. To reduce overfitting and account for the limited number of events, variables with a univariable *p*-value <0.20 were selected for inclusion in the multivariable model. Adjusted associations were then estimated using penalized (Firth) Cox proportional hazards regression. Likewise, natural log transformations were applied to creatinine and BUN to improve model fit. Assessment of the proportional hazards assumption revealed no major violations.

The clinical utility of the Segev model C score for predicting mortality in dogs and cats undergoing hemodialysis was evaluated. Diagnostic accuracy was assessed using Receiver Operating Characteristic (ROC) analysis (8). The diagnostic accuracy for the entire cohort, the ACKD group, and the AKI group was evaluated based on the area under the curve (AUC). The predictive value was considered inadequate with an AUC of <0.70, acceptable with an AUC of 0.70–0.80, excellent with an AUC of 0.80–0.90, and outstanding with an AUC of >0.90 (12).

## Results

### Descriptive statistics and grouping details

A total of 136 animals were included, comprising 67 dogs and 69 cats. Based on the predefined criteria, 66 animals were classified into the ACKD group and 70 into the AKI group. There were no significant differences between two groups in sex or body weight. ACKD group was significantly older than the AKI group (10.39 ± 3.87 years vs. 6.32 ± 3.32 years; *p* < 0.0001). This difference remained significant after Bonferroni correction (adjusted *p* < 0.0001). A significant difference was also observed in serum creatinine (mg/dL) at discharge between two groups (ACKD group, 3.38 ± 1.16 vs. AKI group, 2.81 ± 2.92; *p* = 0.0008; adjusted *p* = 0.0073).

**Figure 1:**
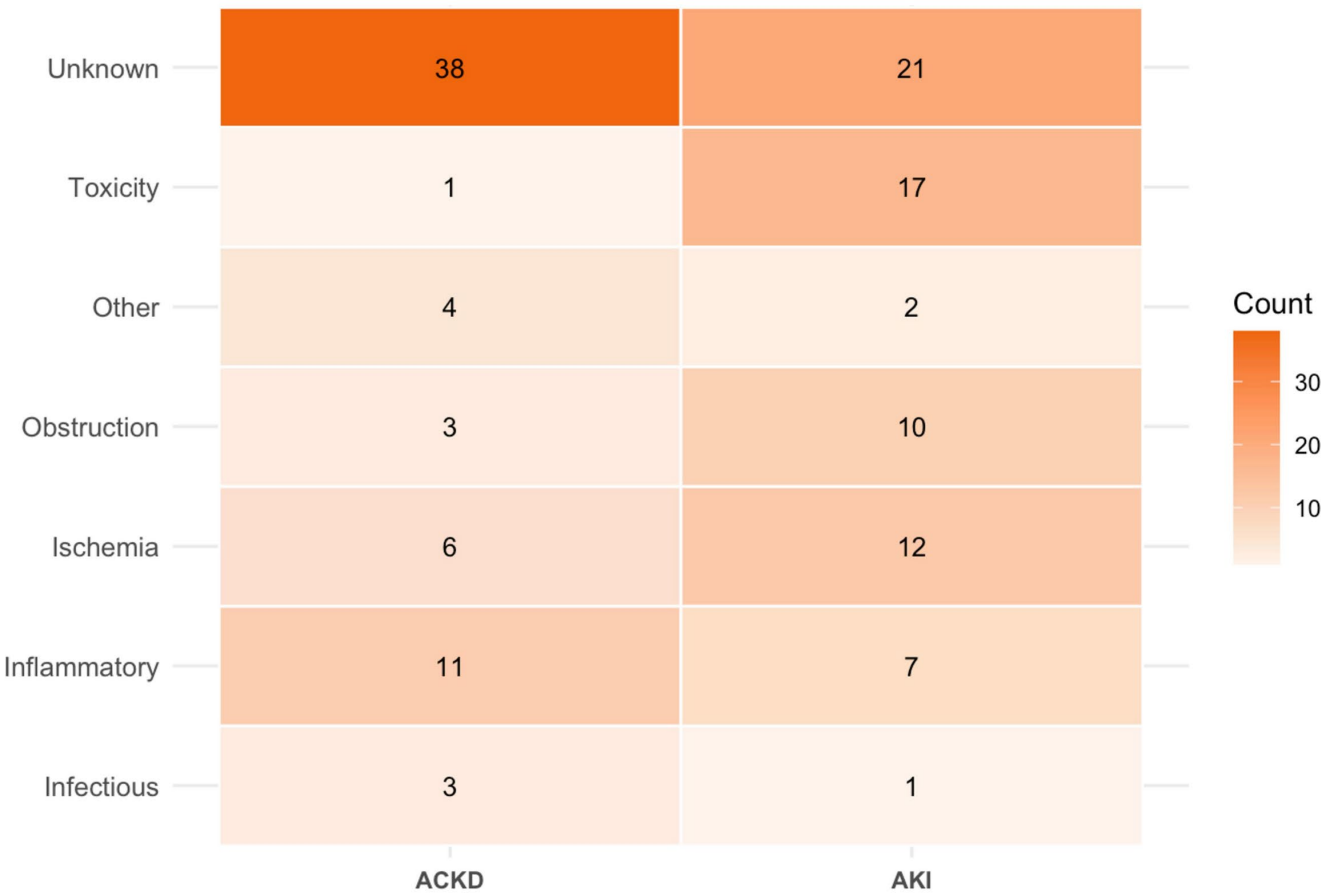
Heatmap of etiologic distribution in acute-on-chronic kidney disease (ACKD) (*n* = 66) and acute kidney injury (AKI) (*n* = 70) groups.

The presumptive causes also differed significantly between ACKD and AKI group (*p* < 0.001). Toxicity was more common in the AKI group, whereas cases of unknown origin were relatively more common in the ACKD group (Figure 1). In contrast, no significant differences were identified in creatinine, BUN, phosphorus at presentation, urine output, or Segev model C. In addition, length of hospitalization and the number of hemodialysis did not differ significantly between the groups. Detailed demographic data, laboratory findings, and treatment-related information are presented in Table 1. Urea reduction ratio ([predialysis urea concentration − postdialysis urea concentration]/predialysis urea concentration) × 100) from each treatment up to fifth treatments were similar between AKI and ACKD (Table 2).

**Table 1:** Baseline characteristics of dogs and cats with acute-on-chronic kidney disease (ACKD) and acute kidney injury (AKI) treated with renal replacement therapy.

Characteristic	ACKD group			AKI group			*p* value	Adjusted *p* value
	Survivors	Non-survivors	Total	Survivors	Non-survivors	Total		
Age	10.76 ± 3.40	9.97 ± 4.37	10.39 ± 3.87	6.06 ± 3.37	6.94 ± 3.21	6.32 ± 3.32	<0.0001	<0.0001
Sex	M (0), MN (23), F (1), SF (11)	M (3), MN (16), F (3), SF (9)	M (3), MN (39), F (4), SF (20)	M (3), MN (31), F (1), SF (13)	M (2), MN (8), F (0), SF (11)	M (5), MN (39), F (1), SF (24)		
Weight	5.59 ± 2.47	5.06 ± 2.31	5.34 ± 2.39	6.84 ± 6.63	5.85 ± 2.34	6.55 ± 5.69	0.3041	1.0000
Species	Canine (15), Feline (20)	Canine (16), Feline (15)	Canine (31), Feline (35)	Canine (24), Feline (25)	Canine (12), Feline (9)	Canine (36), Feline (34)		
Creatinine at presentation	10.99 ± 5.30	11.92 ± 8.56	11.43 ± 6.98	11.08 ± 7.59	14.62 ± 10.10	12.14 ± 8.50	0.7572	1.0000
Creatinine at discharge	3.32 ± 1.13	5.10	3.38 ± 1.16	2.81 ± 2.92	NA	2.81 ± 2.92	0.0008	0.0073
BUN at presentation	145.86 ± 59.23	169.81 ± 83.05	157.11 ± 71.86	146.43 ± 65.60	193.43 ± 103.26	160.53 ± 80.96	0.7374	1.0000
Phosphorus at presentation	13.10 ± 7.09	16.81 ± 7.62	14.84 ± 7.52	12.91 ± 5.19	16.66 ± 8.90	14.04 ± 6.69	0.5295	1.0000
Urine output at presentation	A (14), O (7), N (10), P (2), U (2)	A (13), O (9), N (3), P (3), U (3)	A (27), O (16), N (13), P (5), U (5)	A (20), O (10), N (10), P (5), U (4)	A (13), O (5), N (1), P (1), U (1)	A (33), O (15), N (11), P (6), U (5)		
Segev model C	21.44 ± 4.59	22.20 ± 3.35	21.83 ± 3.96	19.89 ± 4.25	23.64 ± 3.60	21.22 ± 4.37	0.5480	1.0000
Etiology	Infectious (2), Inflammatory (7), Ischemia (2), Obstruction (1), Toxicity (1), Other (3), Unknown (19)	Infectious (1), Inflammatory (4), Ischemia (4), Obstruction (2), Toxicity (0), Other (1), Unknown (19)	Infectious (3), Inflammatory (11), Ischemia (6), Obstruction (3), Toxicity (1) Other (4), Unknown (38)	Infectious (1), Inflammatory (4), Ischemia (9), Obstruction (7), Toxicity (11) Other (2), Unknown (15)	Infectious (0), Inflammatory (3), Ischemia (3), Obstruction (3), Toxicity (6) Other (0), Unknown (6)	Infectious (1), Inflammatory (7), Ischemia (12), Obstruction (10), Toxicity (17) Other (2), Unknown (21)	<0.001	
Length of hospitalization (days) [IQR]	10 [7.5–15.5]	4 [2–6]	7.5 [3.25–11]	10 [7–16]	2 [2–4]	8 [3–11]	0.8036	1.0000
Number of hemodialysis [IQR]	3 [2.5–5]	2 [1–4]	3 [2–4.75]	3 [2–4]	2 [1–2]	2 [2–4]	0.1260	1.0000

**Table 2:** Urea reduction ratio (%) during dialysis sessions in dogs and cats with acute-on-chronic kidney disease (ACKD) and acute kidney injury (AKI) treated with renal replacement therapy.

Dialysis session	Total		ACKD		AKI		*p*
	*n*	Mean ± SD	*n*	Mean ± SD	*n*	Mean ± SD	
1st	130	44 ± 19	66	46 ± 18	64	43 ± 20	0.436
2nd	102	48 ± 14	51	51 ± 9	51	46 ± 17	0.068
3rd	71	50 ± 13	40	52 ± 11	31	47 ± 14	0.114
4th	48	47 ± 18	29	50 ± 14	19	44 ± 23	0.281
5th	29	57 ± 17	18	59 ± 18	11	53 ± 16	0.393

Urea reduction ratio (URR) was calculated as ([predialysis urea concentration − postdialysis urea concentration]/predialysis urea concentration) × 100.

ACKD, Acute-on-chronic kidney disease; AKI, acute kidney injury; SD, standard deviation.

### Short-term survival at discharge

#### Survival at discharge between groups

The short-term survival rate to discharge among all dogs and cats that underwent hemodialysis was 62% (84/136). Of these, the short-term survival rate was 53% (35/66) in the ACKD group and 70% (49/70) in the AKI group. Within the ACKD group, the short-term survival rate was 48% (15/31) in dogs and 57% (20/35) in cats. Within the AKI group, the corresponding rates were 67% (24/36) in dogs and 74% (25/34) in cats, indicating higher survival rates in cats than in dogs in both groups. In the ACKD group, the RMST for short-term survival during hospitalization (≤27 days) was 15 days (95% CI, 11.7–17.7), which was shorter than that in the AKI group (19 days; 95% CI, 16.6–21.1). Kaplan–Meier analysis showed no statistically significant difference in short-term in-hospital survival between the ACKD and AKI groups (log-rank *p* = 0.089), although the survival curves showed modest separation.

#### Analysis of factors associated with survival at discharge

To evaluate factors associated with short-term survival, univariable followed by multivariable logistic regression analyses were performed. In the ACKD group, neuter status and length of hospitalization were significantly associated with survival to discharge (Supplementary Table 1). Based on the candidate variables for multivariable analysis (*p* < 0.20), normal urine output also showed a potential association with survival (OR = 2.79; 95% CI, 0.72–13.07; *p* = 0.141). In contrast, age, sex, body weight, species, creatinine at presentation, BUN at presentation, oliguria, polyuria, Segev model C, and number of hemodialysis were not significantly associated with short-term survival.

In the AKI group, Segev model C, length of hospitalization, and number of hemodialysis sessions were significantly associated with survival to discharge. For each one-point increase in Segev model C, the odds of survival decreased by 20%, indicating a lower likelihood of survival with increasing score (OR = 0.80; 95% CI, 0.62–0.96; *p* = 0.016). In addition, each one-day increase in length of hospitalization was associated with a 1.57-fold increase in the odds of survival (OR = 1.57; 95% CI, 1.28–2.03; *p* < 0.0001), and each additional hemodialysis session was associated with a 1.99-fold increase in the odds of survival (OR = 1.99; 95% CI, 1.25–3.60; *p* = 0.001). Candidate variables selected for multivariable analysis included creatinine at presentation (OR = 0.54; 95% CI, 0.24–1.07; *p* = 0.078), BUN at presentation (OR = 0.39; 95% CI, 0.12–1.05; *p* = 0.065), and normal urine output relative to anuria (OR = 4.61; 95% CI, 0.91–46.26; *p* = 0.067). The remaining variables, including age, sex, neuter status, body weight, species, oliguria, and polyuria, were not significantly associated with short-term survival.

In the subsequent multivariable logistic regression analysis, only length of hospitalization remained independently associated with survival to discharge in the ACKD group (OR, 1.21; 95% CI, 1.09–1.39; *p* = 0.0002), whereas no independent predictor was identified in the AKI group (Supplementary Table 2).

### Long-term survival analysis

#### Comparison of long-term prognosis among animals that survived

**Figure 2:**
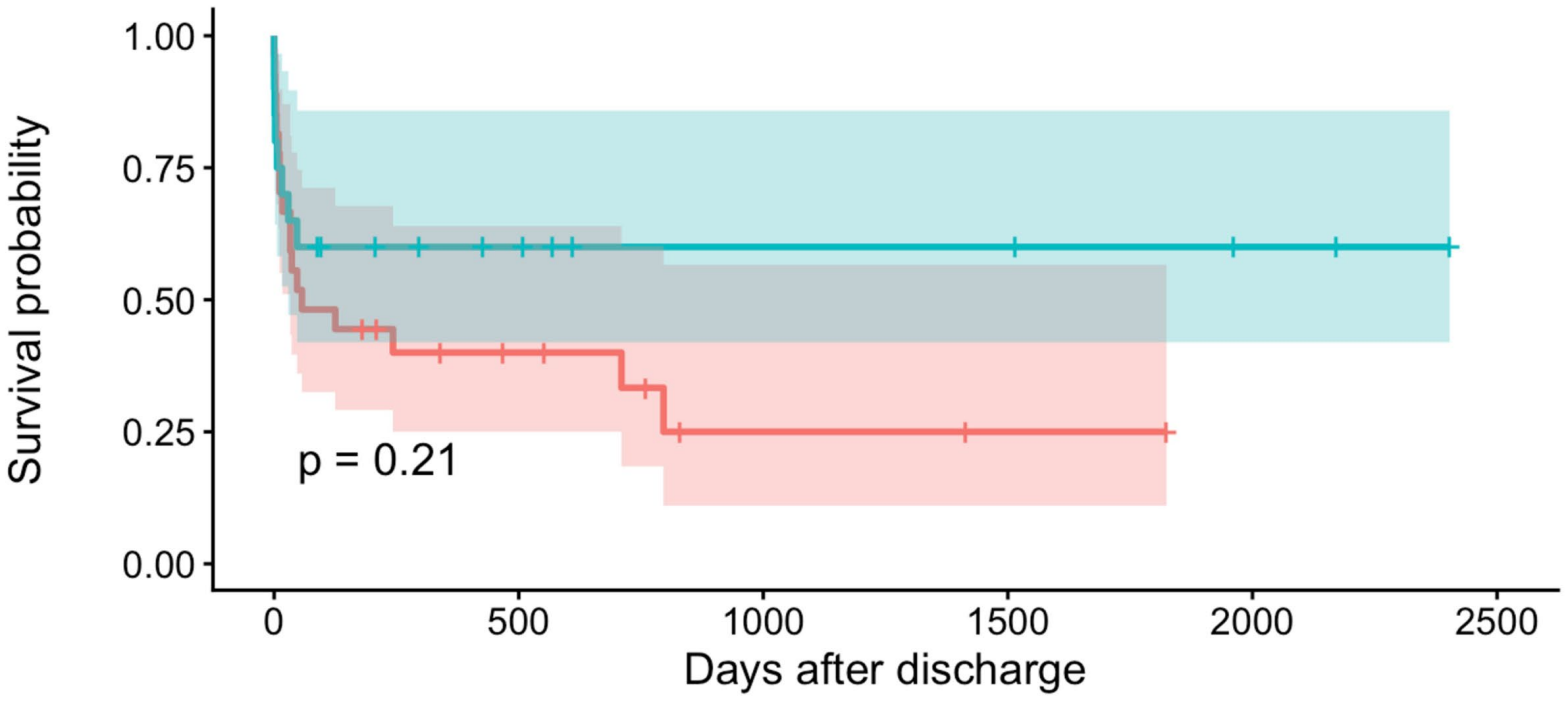
Kaplan–Meier survival curves for comparison of long-term survival between acute-on-chronic kidney disease (ACKD) and acute kidney injury (AKI) group. Red: ACKD group; Blue: AKI groups. ACKD: Median: 57d; RMST (<=1824d) = 594.9 (95% CI: 280.7909.2). AKI: Median: Not reached d; RMST (<=1824d) = 1099.5 (95% CI: 710.5–1488.4).

Among all dogs and cats that survived to discharge, long-term outcome was evaluated. The median survival time for the ACKD group was 57 days; specifically, the median survival time for dogs was 33 days. The median survival time for cats with ACKD could not be determined because more than 50% were still alive 2,400 days after discharge. The RMST for long-term survival (≤1824 days) was 594.9 days (95% CI, 280.7–909.2) in the ACKD group, which was shorter than that in the AKI group (1099.5 days; 95% CI, 710.5–1488.4). Likewise, the Kaplan–Meier survival curves showed that the ACKD group consistently tended to have lower survival probabilities over time than the AKI group (Figure 2). However, despite this trend, log-rank testing indicated that the difference in long-term survival between the ACKD and AKI groups was not statistically significant (*p* = 0.21).

#### Analysis of factors associated with long-term prognosis

To evaluate factors associated with long-term outcome, univariable Cox proportional hazards analyses were performed, and the results are presented in Supplementary Table 3.

In the ACKD group, age, species, creatinine at discharge, and urine output at presentation were significantly associated with long-term survival. Increasing age was associated with an increased hazard of death (HR = 1.18; 95% CI, 1.00–1.40; *p* = 0.044), and species was also significantly associated with long-term survival (HR = 0.31; 95% CI, 0.11–0.89; *p* = 0.030). In addition, higher log-transformed creatinine at discharge was associated with an increased hazard of death (HR = 6.63; 95% CI, 1.16–38.00; *p* = 0.034), and urine output at presentation was also significantly associated with poorer long-term outcome (HR = 5.92; 95% CI, 1.42–24.8; *p* = 0.015). Although log-transformed creatinine at presentation did not reach statistical significance, it showed a potential association with long-term outcome and was therefore considered a candidate variable for multivariable analysis (HR = 0.41; 95% CI, 0.15–1.11; *p* = 0.080).

In the AKI group, body weight was the only variable significantly associated with long-term survival. Higher body weight was associated with an increased hazard of death (HR = 1.39; 95% CI, 1.02–1.88; *p* = 0.036). Although not statistically significant, log-transformed creatinine at discharge met the criterion for inclusion as a candidate variable in the multivariable analysis (HR = 80.03; 95% CI, 0.2–32264.91; *p* = 0.152). In contrast, age, neuter status, species, creatinine at presentation, BUN at presentation, urine output at presentation, Segev model C, length of hospitalization, and number of hemodialysis were not significantly associated with long-term survival in the AKI group.

In the multivariable Cox proportional hazards analysis Supplementary Table 4, only urine output at presentation remained independently associated with long-term survival in the ACKD group. Interestingly, relative to anuria, normal urine output was significantly associated with a higher hazard of poor long-term outcome (HR = 5.26; 95% CI, 1.25–33.56; *p* = 0.022). In contrast, no independent predictor was identified in the AKI group.

### Application of Segev score to ACKD and AKI group for predicting survival to discharge

When the analysis was performed in the overall cohort (*n* = 65) included in the ROC analysis, the AUC for the Segev score was 0.73 (95% CI, 0.61–0.85), indicating overall acceptable discriminative ability. However, when the cohort was stratified into the two groups, a clear difference in predictive performance was observed. In the AKI group (*n* = 33), the AUC was 0.84 (95% CI, 0.70–0.98), indicating excellent performance for predicting short-term survival. In contrast, in the ACKD group (*n* = 32), the AUC was only 0.59 (95% CI, 0.38–0.81), indicating inadequate mortality prediction.

## Discussion

This study compared outcomes between dogs and cats with *de novo* AKI and those with ACKD treated with RRT. Three main findings were identified. First, compared with the AKI group, the ACKD group was significantly older (10.4 ± 3.9 vs. 6.3 ± 3.3 years; *p* < 0.0001) and had a higher serum creatinine at discharge (3.4 ± 1.2 vs. 2.8 ± 2.9; adjusted *p* = 0.0073), whereas creatinine, BUN, phosphorus, urine output, and Segev model C score at presentation did not differ significantly between the two groups. These findings suggest that, although the severity of illness at presentation was similar between groups, renal recovery by discharge was less complete as anticipated in the ACKD group. Second, contrary to our hypothesis, survival to discharge was lower in the ACKD group than in the AKI group (53% vs. 70%). Kaplan–Meier analysis during hospitalization likewise showed a trend toward poorer short-term survival in the ACKD group, although the difference did not reach statistical significance (*p* = 0.089). Nevertheless, even among patients requiring hemodialysis, the ACKD group still showed a clinically meaningful short-term survival rate. Third, among patients that survived to discharge, those in the ACKD group had numerically shorter long-term survival and a lower RMST than those in the AKI group, although this difference was not statistically significant (*p* = 0.21). Taken together, these findings describe the clinical outcomes of RRT-treated ACKD patients and suggest that underlying CKD may adversely affect both immediate outcome and the durability of recovery after discharge.

Notably, a significant etiologic difference was observed between ACKD and *de novo* AKI in the present study. To maintain consistency and comparability with previous ACKD studies in dogs and cats, the etiologic categories used in those studies were adopted in the present study. ACKD group was characterized by a markedly higher proportion of cases with unknown etiology (57.6%), compared to AKI group (30%, 21/70). This finding is consistent with previous ACKD reports in dogs (45%) and cats (66%) (5, 6). Taken together, these findings suggest that ACKD reflects a more complex and heterogeneous decompensated state superimposed on chronic renal injury.

In the present study, inflammatory causes (such as pancreatitis or glomerulopathies), toxicities, and infections constituted a major portion of the underlying etiologies. It is important to note that conditions like severe pancreatitis or sepsis act as complex systemic comorbidities rather than isolated renal insults. For instance, pancreatitis induces AKI through a combination of severe hypovolemia and systemic inflammatory response syndrome-mediated renal microvascular endothelial injury. These systemic inflammatory processes, compounded by uremia-induced thrombocytopathy or inflammation-driven coagulopathies, can independently and severely jeopardize patient survival regardless of the efficacy of RRT. Although we attempted to mitigate this confounding effect by adjusting for baseline clinical severity using the Segev model C score in our statistical models, the potential influence of these diverse, non-renal systemic pathologies on the clinical course should be carefully considered when interpreting the survival outcomes.

Previous ACKD studies in dogs and cats found that short-term outcomes were comparable between AKI and ACKD patients (5, 6). In the present study, the discharge survival rate in the ACKD group was 53%, which was lower than the 65% reported in dogs and the 58% reported in cats in previous ACKD studies. An important distinction, however, is that those earlier studies evaluated broader canine and feline populations, including less severe cases that recovered with medical management alone. By contrast, the present study was restricted to patients with kidney injury severe enough to require RRT. In this setting, the lower discharge survival rate in ACKD patients suggests that the burden of underlying CKD has a more direct influence on prognosis. Importantly, however, 53% of ACKD patients still survived to discharge, indicating that RRT should not be regarded as futile for patients with ACKD. Rather, ACKD should be viewed as a condition associated with a more guarded short-term prognosis than AKI, while still retaining a meaningful likelihood of survival to discharge. Meanwhile, 70% of the AKI cohort of the present study survived to discharge which was higher than that reported in previous study of dogs (53%) and cats (50%) (12, 13). Although direct comparison is limited, this difference is likely attributable to differences in study period, case selection, and treatment modality (PIRRT vs. IHD).

With regard to short-term prognostic factors, after multivariable analysis, length of hospitalization was the only independent variable significantly associated with survival to discharge in the ACKD group, whereas no independent predictor remained significant in the AKI group. These findings should be interpreted cautiously. Length of hospitalization is unlikely to represent a true prognostic factor; rather, it more likely reflects survivor bias, as patients survived long enough to receive ongoing supportive care and demonstrate signs of renal recovery. In other words, length of hospitalization should be viewed not as a prognostic predictor, but as a course-related variable that tends to be longer among survivors. The number of hemodialysis, which was associated with short-term survival in the univariable analysis in the AKI group but lost significance after multivariable analysis, may be interpreted similarly. Accordingly, these variables should not be interpreted as indicating that dialysis treatment or prolonged hospitalization itself causes survival, but rather that patients who survived the early critical period and continued to receive hemodialysis had a greater opportunity for recovery.

In the present study, median survival time after discharge was 57 days in dogs and cats with ACKD, specifically 33 days in dogs. Kaplan–Meier curves and RMST suggested persistently lower survival probabilities in ACKD group over time. Even without statistical significance, this trend is clinically important as it suggests that the effect of underlying CKD extends beyond the initial hospitalization. The lack of statistical significance was likely influenced by the limited sample size and censoring. Although direct comparison is limited, this trend is broadly consistent with previous reports showing median survival times of 105 days in canine ACKD and 66 days in feline ACKD, in contrast to 1,322 days in dogs recovering from *de novo* AKI (5, 6, 14). Taken together, these findings indicate that some ACKD patients treated with RRT survived the acute crisis to discharge, whereas the long-term trajectory after discharge remains strongly affected by the progression of underlying CKD, incomplete renal recovery from acute injury, and subsequent post-discharge complications.

In the ACKD group, only urine output remained significant in the multivariable model, with normuria being associated with a higher risk of death than anuria. This finding is not physiologically intuitive and contrasts with previous studies that have considered urine production a potential marker of recovery (13, 15, 16). Given the small sample size, limited number of events, and wide confidence intervals in the Cox analysis, this result is best viewed as an exploratory signal. One possible explanation is that, in CKD patients, apparently normal urine output does not necessarily indicate preserved renal reserve, but may instead reflect impaired concentrating ability, chronic tubulointerstitial damage, or non-oliguric decompensation. In addition, urine output at presentation may have been influenced by prior fluid therapy and diuretic exposure. Because these treatments were inconsistently documented in referred cases, adjustment for these variables was not feasible. One possible explanation is that urine output in ACKD may reflect the relative contribution of acute versus chronic injury. Patients with a more prominent acute component may be more likely to present with oliguria or anuria, whereas patients with a greater chronic component may maintain apparently normal urine output despite substantial loss of renal functional reserve. Overall, these findings suggest that the prognostic value of urine output in ACKD may differ from its interpretation in *de novo* AKI.

The relationship between creatinine at discharge and long-term survival is also noteworthy. In the ACKD group, higher discharge creatinine was associated with poorer long-term outcome in the univariable analysis and showed a strong directional effect in the multivariable model, although it did not reach statistical significance. This finding is consistent with the feline ACKD study by Chen et al., in which creatinine at discharge was also identified as a factor associated with long-term outcome (6). This result is clinically intuitive, as creatinine at discharge may serve as a surrogate marker for the degree of residual renal dysfunction after the acute episode. In other words, among ACKD patients that survived hospitalization, the extent of renal recovery achieved by the time of discharge may have a greater influence on subsequent survival than the severity of creatinine at presentation. In contrast, no stable independent predictor of long-term outcome was identified among AKI survivors, which may reflect the smaller number of past injury events.

Another clinically relevant finding was the predictive performance of the Segev model C (8). Overall, the model showed acceptable discrimination in entire cohorts and excellent in AKI group, but its discriminative ability was inadequate in ACKD group. Consistent with this, univariable analysis showed that higher Segev model C scores were associated with lower short-term survival only in the AKI group (OR = 0.80; 95% CI, 0.62–0.96; *p* = 0.016), not in the ACKD group. This suggests that a prognostic scoring system developed for AKI may retain clinical utility in dogs and cats with *de novo* AKI requiring RRT, but may perform less well when applied to those with AKI and concurrent underlying CKD. This is not unexpected as prognosis in ACKD is determined not only by the severity of the acute insult, but also by chronic nephron loss, baseline renal reserve, and the ability to recover to a functionally sustainable state after the crisis (17, 18). Accordingly, our findings suggest that ACKD-specific prognostic tools may be needed. Ideally, future models should incorporate variables reflecting both acute severity and chronic renal burden, such as baseline creatinine, CKD stage, diagnostic imaging, and biomarkers of renal recovery at discharge.

Because all animals in this cohort received RRT, the present study cannot determine whether RRT improved survival compared with conventional medical management. A previous proportional meta-analysis found no statistically significant difference in mortality between animals treated with dialysis and those managed conservatively (19). Moreover, in a single-center retrospective cohort, identifying an untreated comparison group with equivalent indications for RRT and comparable baseline disease severity is challenging, because the decision not to pursue RRT may be influenced by concurrent disease, clinical instability, owner preference, financial limitations, or transition to palliative care. Such a comparison would therefore be highly susceptible to being confounded by indication. Future multicenter studies using prospectively defined eligibility criteria and severity-matched or appropriately adjusted comparison groups are needed to evaluate the clinical efficacy of RRT compared to medical management alone.

This study has several limitations. First, there are inherent limitations associated with the retrospective, single-center study design. Due to the retrospective nature, comprehensive screening for non-azotemic underlying CKD relied entirely on available historical records. Because urinalysis data or specific tubular injury biomarkers (e.g., urinary Cystatin B) were not uniformly evaluated across all patients prior to referral, a degree of misclassification bias cannot be completely excluded; some early-stage, non-azotemic CKD patients may have been inadvertently categorized into the *de novo* AKI group. Second, the small sample size within subgroups and the substantial amount of censored data in the long-term analyses reduced statistical power and widened confidence intervals. Third, RRT outcomes are influenced by non-biological factors such as owner finances and euthanasia decisions which affect survival independently of disease severity.

## Conclusion

To conclude, this is the first retrospective study comparing outcomes and prognostic factors between ACKD and AKI in dogs and cats with kidney injury severe enough to require hemodialysis. In this study, ACKD was associated with older age, a different etiologic distribution, lower survival to discharge, and shorter long-term survival than AKI group. Still, more than half of the ACKD patients survived to discharge, suggesting that RRT may remain a reasonable supportive option in selected ACKD dogs and cats. These findings suggest that RRT in ACKD should not be dismissed as futile, but rather approached with careful consideration. Larger prospective multicenter studies are needed to improve prognostic assessment and risk stratification.

## Data Availability

The raw data supporting the conclusions of this article will be made available by the authors, without undue reservation.

## References

[1] Rimer D Chen H Bar-Nathan M Segev G. Acute kidney injury in dogs: etiology, clinical and clinicopathologic findings, prognostic markers, and outcome. J Vet Intern Med. (2022) 36:609–18. doi: 10.1111/jvim.16375, 35103347 PMC8965273

[2] Dadousis C Whetton AD Mwacalimba K Merlo A Wright A Geifman N. Renal disease in cats and dogs—lessons learned from text-mined trends in humans. Animals (Basel). (2024) 14:3349. doi: 10.3390/ani14233349 PMC11639467 39682316

[3] Segev G Cortellini S Foster JD Francey T Langston C Londoño L . International renal interest society best practice consensus guidelines for the diagnosis and management of acute kidney injury in cats and dogs. Vet J. (2024) 305:106068. doi: 10.1016/j.tvjl.2024.106068 38325516

[4] Chen H Cowgill LD Francey T Jepson RE Langston C Schweighauser A . International renal interest society best practice consensus guidelines on the use of continuous renal replacement therapy in dogs and cats. Vet J. (2026) 315:106548. doi: 10.1016/j.tvjl.2026.106548, 41513167

[5] Dunaevich A Chen H Musseri D Kuzi S Mazaki-Tovi M Aroch I . Acute on chronic kidney disease in dogs: etiology, clinical and clinicopathologic findings, prognostic markers, and survival. J Vet Intern Med. (2020) 34:2507–15. doi: 10.1111/jvim.15931, 33044036 PMC7694831

[6] Chen H Dunaevich A Apfelbaum N Kuzi S Mazaki-Tovi M Aroch I . Acute on chronic kidney disease in cats: etiology, clinical and clinicopathologic findings, prognostic markers, and outcome. J Vet Intern Med. (2020) 34:1496–506. doi: 10.1111/jvim.15808, 32445217 PMC7379052

[7] Vaden SL Levine J Breitschwerdt EB. A retrospective case-control of acute renal failure in 99 dogs. J Vet Intern Med. (1997) 11:58–64. doi: 10.1111/j.1939-1676.1997.tb00074.x, 9127291

[8] Segev G Langston C Takada K Kass PH Cowgill LD. Validation of a clinical scoring system for outcome prediction in dogs with acute kidney injury managed by hemodialysis. J Vet Intern Med. (2016) 30:803–7. doi: 10.1111/jvim.13930, 26995335 PMC4913579

[9] Giorgi ME Mochel JP Yuan L Adin DB Ward JL. Retrospective evaluation of risk factors for development of kidney injury after parenteral furosemide treatment of left-sided congestive heart failure in dogs. J Vet Intern Med. (2022) 36:2042–52. doi: 10.1111/jvim.16571, 36254646 PMC9708435

[10] Lomas AL Grauer GF. The renal effects of NSAIDs in dogs. J Am Anim Hosp Assoc. (2015) 51:197–203. doi: 10.5326/JAAHA-MS-6239, 25955147

[11] Whelton A. Nephrotoxicity of nonsteroidal anti-inflammatory drugs: physiologic foundations and clinical implications. Am J Med. (1999) 106:13S–24S. doi: 10.1016/S0002-9343(99)00113-8, 10390124

[12] Mandrekar JN. Receiver operating characteristic curve in diagnostic test assessment. J Thorac Oncol. (2010) 5:1315–6. doi: 10.1097/JTO.0b013e3181ec173d, 20736804

[13] Eatroff A Langston C Chalhoub S Poeppel K Mitelberg E. Long-term outcome of cats and dogs with acute kidney injury treated with intermittent hemodialysis: 135 cases (1997-2010). J Am Vet Med Assoc. (2012) 241:1471–8. doi: 10.2460/javma.241.11.1471, 23176239

[14] Bar-Nathan M Chen H Rimer D Segev G. Long-term outcome of dogs recovering from acute kidney injury: 132 cases. J Vet Intern Med. (2022) 36:1024–31. doi: 10.1111/jvim.16435, 35478193 PMC9151474

[15] Segev G Nivy R Kass PH Cowgill LD. A retrospective study of acute kidney injury in cats and development of a novel clinical scoring system for predicting outcome for cats managed by hemodialysis. J Vet Intern Med. (2013) 27:830–9. doi: 10.1111/jvim.12108, 23679089

[16] Thoen ME Kerl ME. Characterization of acute kidney injury in hospitalized dogs and evaluation of a veterinary acute kidney injury staging system. J Vet Emerg Crit Care. (2011) 21:648–57. doi: 10.1111/j.1476-4431.2011.00689.x, 22316258

[17] Rudinsky AJ Harjes LM Byron J Chew DJ Toribio RE Langston C . Factors associated with survival in dogs with chronic kidney disease. J Vet Intern Med. (2018) 32:1977–82. doi: 10.1111/jvim.15322, 30325060 PMC6271312

[18] Cowgill LD Polzin DJ Elliott J Nabity MB Segev G Grauer GF . Is progressive chronic kidney disease a slow acute kidney injury? Vet Clin N Am Small Anim Pract. (2016) 46:995–1013. doi: 10.1016/J.CVSM.2016.06.001, 27593574

[19] Legatti SAM El Dib R Legatti E Botan AG Camargo SEA Agarwal A . Acute kidney injury in cats and dogs: a proportional meta-analysis of case series studies. PLoS One. (2018) 13:e0190772. doi: 10.1371/journal.pone.0190772, 29370180 PMC5784898

