# Supplementary material for: Dogs and cats with acute-on-chronic kidney disease have worse outcomes than those with acute kidney injury following renal replacement therapy
Source: Front Vet Sci. 2026 Jul 30;13:1857111. doi: 10.3389/fvets.2026.1857111 (PMC13475998; doi:10.3389/fvets.2026.1857111)
Supplement: Supplementary file 1 [file Table_1.docx]

Supplementary Table 1. Analysis of factors associated with survival at discharge (short-term) by univariable logistic regression in dogs and cats with acute-on-chronic kidney disease (ACKD) or acute kidney injury (AKI).

| Factors | Sub factors | ACKD group | | | | AKI group | | | |
| --- | --- | --- | --- | --- | --- | --- | --- | --- | --- |
|  |  | N | OR(survival) | 95% CI | P-value | N | OR(survival) | 95% CI | P-value |
| Age |  | 66 | 1.05 | 0.93-1.20 | 0.416 | 70 | 0.92 | 0.79-1.08 | 0.313 |
| Sex  (Reference: Male) | Female | 66 | 3.00 | 0.11-504.52 | 0.521 | 70 | 2.14 | 0.07-369.65 | 0.667 |
| Neutered  (Reference: Intact) | Neutered | 66 | 5.86 | 1.13-58.82 | **0.034**** | 69 | 1.27 | 0.21-6.27 | 0.778 |
| Weight |  | 66 | 1.09 | 0.90-1.36 | 0.385 | 70 | 1.02 | 0.94-1.17 | 0.681 |
| Species  (Reference: Canine) |  | 66 | 1.41 | 0.54-3.71 | 0.483 | 70 | 1.37 | 0.50-3.83 | 0.540 |
| Creatinine at presentation |  | 66 | 1.04 | 0.46-2.39 | 0.920 | 70 | 0.54 | 0.24-1.07 | 0.078* |
| BUN at presentation |  | 66 | 0.56 | 0.18-1.55 | 0.266 | 70 | 0.39 | 0.12-1.05 | 0.065* |
| Urine output at presentation  (Reference: Anuria) | Oligouria | 61 | 0.74 | 0.21-2.46 | 0.618 | 65 | 1.26 | 0.37-4.57 | 0.716 |
|  | Normouria | 61 | 2.79 | 0.72-13.07 | 0.141* | 65 | 4.61 | 0.91-46.26 | 0.067* |
|  | Polyuria | 61 | 0.67 | 0.10-3.98 | 0.652 | 65 | 2.41 | 0.41-25.48 | 0.344 |
| Segev model C |  | 33 | 0.96 | 0.80-1.13 | 0.596 | 34 | 0.80 | 0.62-0.96 | **0.016**** |
| Length of hospitalization |  | 66 | 1.22 | 1.10-1.39 | **<0.0001**** | 70 | 1.57 | 1.28-2.03 | **<0.0001**** |
| Number of hemodialysis |  | 66 | 1.12 | 0.93-1.40 | 0.235 | 70 | 1.99 | 1.25-3.60 | **0.001**** |

** P<0.05; *P<0.2 for multivariable logistic regression test

Supplementary Table 2. Analysis of factors associated with survival at discharge (short-term) by multivariable logistic regression in dogs and cats with acute-on-chronic kidney disease (ACKD) or acute kidney injury (AKI).

| Factors | ACKD group | | | AKI group | | |
| --- | --- | --- | --- | --- | --- | --- |
|  | OR (survival) | 95% CI | P-value | OR (survival) | 95% CI | P-value |
| Length of hospitalization | 1.21 | 1.09-1.39 | **0.0002**** | 1.18 | 0.97-1.64 | 0.1036 |
| Number of hemodialysis | - | - | - | 1.24 | 0.76-2.41 | 0.4008 |
| Segev model C | - | - | - | 0.90 | 0.67-1.15 | 0.4168 |
| BUN at presentation | - | - | - | 0.84 | 0.11-5.07 | 0.8508 |
| Neutered (Reference: Intact) | 4.27 | 0.76-44.49 | 0.1028 | - | - | - |

Supplementary Table 3. Analysis of risk factors associated with long-term survival by univariable Cox proportional hazards models in dogs and cats with acute-on-chronic kidney disease (ACKD) or acute kidney injury (AKI).

|  | ACKD group | | | | AKI group | | | |
| --- | --- | --- | --- | --- | --- | --- | --- | --- |
| Predictor | N | HR | 95% CI | P-value | N | HR | 95% CI | P-value |
| Age | 26 | 1.18 | 1.00-1.40 | **0.044**** | 18 | 1.15 | 0.89-1.50 | 0.292 |
| Sex | 26 | 2.34 | 0.26-20.93 | 0.448 | 18 | NA | NA | NA |
| Neutered | 26 | 0.51 | 0.07-4.01 | 0.524 | 18 | 0.67 | 0.08-5.74 | 0.712 |
| Weight | 26 | 1.04 | 0.79-1.37 | 0.770 | 18 | 1.39 | 1.02-1.88 | **0.036**** |
| Species | 26 | 0.31 | 0.11-0.89 | **0.030**** | 18 | 0.34 | 0.06-1.88 | 0.217 |
| log(creatinine at presentation) | 26 | 0.41 | 0.15-1.11 | **0.080*** | 18 | 0.99 | 0.43-2.28 | 0.972 |
| log(creatinine at discharge) | 21 | 6.63 | 1.16-38.00 | **0.034**** | 14 | 80.03 | 0.2-32264.91 | **0.152** |
| log(BUN at presentation) | 26 | 1.13 | 0.31-4.10 | 0.852 | 18 | 0.93 | 0.19-4.65 | 0.929 |
| Urine output at presentation | 17 | 5.92 | 1.42-24.8 | **0.015**** | 17 | 0.00 | 0.00-Infinite | 0.999 |
| Segev model C | 14 | 0.99 | 0.84-1.16 | 0.901 | 9 | 0.93 | 0.62-1.39 | 0.715 |
| Length of hospitalization | 26 | 0.95 | 0.85-1.05 | 0.292 | 18 | 0.94 | 0.82-1.09 | 0.423 |
| Number of hemodialysis | 26 | 1.01 | 0.81-1.26 | 0.920 | 18 | 0.97 | 0.74-1.28 | 0.847 |

HR, hazard ratio; CI, confidence interval

Supplementary Table 4. Analysis of risk factors associated with long-term survival by multivariable Cox proportional hazards models in dogs and cats with acute-on-chronic kidney disease or acute kidney injury.

|  | | ACKD group | | | AKI group | | |
| --- | --- | --- | --- | --- | --- | --- | --- |
| Predictor | | HR | 95% CI | P-value | HR | 95% CI | P-value |
| Age |  | 1.01 | 0.68-1.53 | 0.977 |  |  |  |
| Weight |  |  |  |  | 0.98 | 0.59-1.48 | 0.910 |
| Species  (Reference: Canine) |  | 1.44 | 0.17-13.61 | 0.740 |  |  |  |
| Urine output at presentation  (Reference: Anuria) | Oligouria | 6.43 | 0.45-101.03 | 0.165 |  |  |  |
|  | Normouria | 5.26 | 1.25-33.56 | **0.022**** |  |  |  |
|  | Polyuria | 3.74 | 0.30-35.93 | 0.269 |  |  |  |
| log(creatinine at presentation) |  | 0.64 | 0.03-20.88 | 0.794 |  |  |  |
| log(creatinine at discharge) |  | 5.97 | 0.75-98.01 | 0.095 | 19.36 | 0.60-16135.42 | 0.119 |

HR, hazard ratio; CI, confidence interval
